# Development﻿ of fully automated anterior chamber cell analysis based on image software

**DOI:** 10.1038/s41598-021-89794-0

**Published:** 2021-05-21

**Authors:** Tae Seen Kang, Yeongseop Lee, Seongjin Lee, Kyonghoon Kim, Woong-sub Lee, Woohyuk Lee, Jin Hyun Kim, Yong Seop Han

**Affiliations:** 1 Department of Ophthalmology, Gyeongsang National University Changwon Hospital, #11 Samjeongja-ro, Seongsan-gu, Changwon, 51472 Republic of Korea; 2 Department of AI Convergence Engineering, Gyeongsang National University, Jinju, Republic of Korea; 3 School of Computer Science and Engineering, Kyungpook National University, Daegu, Republic of Korea; 4 Department of Information and Communication Engineering, Gyeongsang National University, Tongyeong, Republic of Korea; 5 Department of Ophthalmology, Gyeongsang National University College of Medicine, Jinju, Republic of Korea; 6 Health Science Institute, Gyeongsang National University, Jinju, Republic of Korea

**Keywords:** Corneal diseases, Uveal diseases

## Abstract

Optical coherence tomography (OCT) is a noninvasive method that can quickly and accurately examine the eye at the cellular level. Several studies have used OCT for analysis of anterior chamber cells. However, these studies have several limitations. This study was performed to supplement existing reports of automated analysis of anterior chamber cell images using spectral domain OCT (SD-OCT) and to compare this method with the Standardization of Uveitis Nomenclature (SUN) grading system. We analyzed 2398 anterior segment SD-OCT images from 34 patients using code written in Python. Cell density, size, and eccentricity were measured automatically. Increases in SUN grade were associated with significant cell density increases at all stages (p < 0.001). Significant differences were observed in eccentricity in uveitis, post-surgical inflammation, and vitreous hemorrhage (p < 0.001). Anterior segment SD-OCT is reliable, fast, and accurate means of anterior chamber cell analysis. This method showed a strong correlation with the SUN grade system. Also, eccentricity could be helpful as a supplementary evaluation tool.

## Introduction

The anterior chamber of the eye is the space between the iris and the cornea, which is generally transparent and has no blood cells. However, in many ophthalmological diseases, the normal blood–aqueous barrier is disrupted and blood cells may appear in the anterior chamber. For example, red blood cells (RBCs) appear in the anterior chamber in hyphema, and white blood cells (WBCs) appear in the anterior chamber in uveitis. In endophthalmitis and toxic anterior segment syndrome, the anterior chamber becomes cloudy with the presence of large numbers of WBCs due to leakage of protein and plasma.

The Standardization of Uveitis Nomenclature (SUN) grading system is widely considered the standard method to evaluate pathological changes in the anterior chamber^1^. The method involves assigning grades from 0 to 4 by counting the number of cells using a 1 × 1-mm slit beam in the anterior chamber. However, the SUN grading system has disadvantages, including variation in the results between examiners^2,3^ and it is non-quantitative. In addition, the grades may vary depending on the intensity and area of light^4^, and an increase in the number of blood cells may lead to extended examination and inaccurate results^3^. As the intervals between grades are not equal, each grade of improvement may indicate a clinically different degree of improvement. There is a limitation in that severe inflammation over grade 3+ is equally rated as grade 4+ regardless of clinical deterioration or improvement. Therefore, it is difficult to quantify therapeutic effects objectively based on SUN grading system.

Optical coherence tomography (OCT) is a noninvasive method that can quickly and accurately examine the eye at the cellular level. As it has been widely used for diagnosis of diseases of the retina and optic disc, it is readily accessible by ophthalmologists. In addition, the anterior segment can be examined using only an add-on lens. Similar to retina imaging, the anatomical structure of the anterior segment can be identified in a noninvasive manner and at very high resolution at the cellular level. Due to these advantages, several studies have used OCT for analysis of anterior chamber cells^5–14^.

This study was performed to supplement existing work. We analyzed anterior chamber cells using spectral-domain OCT (SD-OCT) images, developed an automated program, and compared with existing SUN grading system. The proposed method allows quantitative evaluation of the anterior chamber cells both rapidly and accurately.

## Results

### Baseline characteristics

In this study, 34 medical records were analyzed retrospectively. The study population consisted of 27 men and seven women with an average age of 57.5 ± 12.2 years. Of the 34 patients, 27 had post-surgical inflammation, six had uveitis, and one had vitreous hemorrhage. In total, 2415 images were obtained from these subjects. After excluding 17 images in which we could not distinguish air, cornea, and anterior chamber, we analyzed 2398 images. In total, 1756 images were from examinations of eyes in which cells were currently or had previously been seen in the anterior chamber under slit lamp examination, while 642 images were of the opposite eye, which was clinically normal. In the medical records, 896 images were classified as SUN grade 0+ or normal, 829 as 1/2+, 242 as 1+, 168 as 2+, 189 as 3+, and 74 as 4+.

### Comparison of cell density by SUN grade

**Figure 1:**
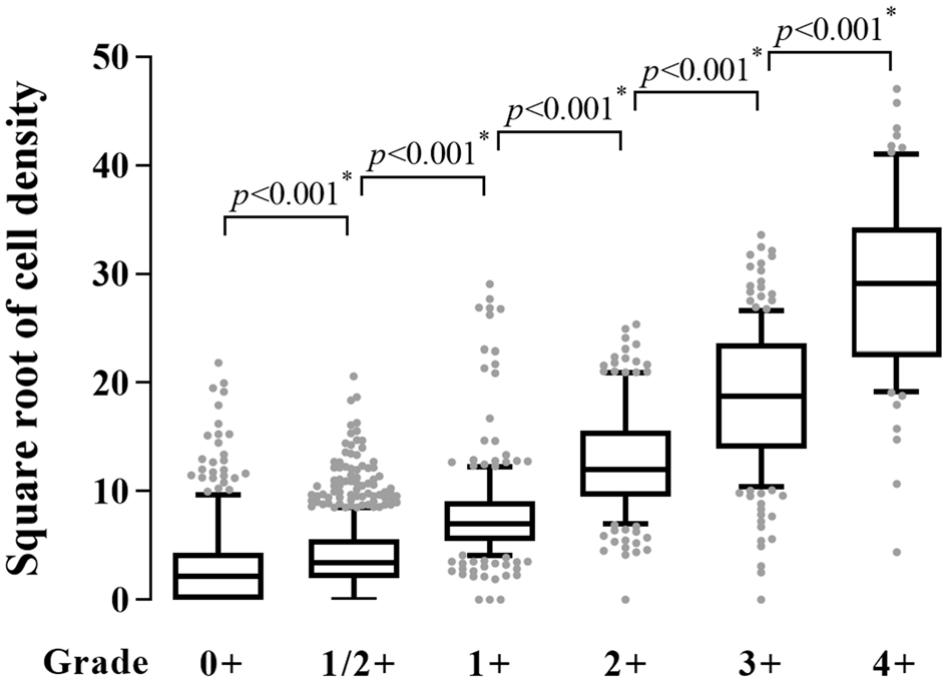
The square root of cell density according to SUN grade. Square root transformation was performed because the intervals between SUN grades are not equal, and the intervals increase with increasing grade. The cell density also increased significantly with increasing SUN grade. The boxes indicate the median and interquartile range, and the whiskers indicate 10–90 percentile.

Cell density (number of cells per million pixels) of SD-OCT image according to clinical SUN grade are shown in Table 1. Increases in SUN grade were associated with significant increases in cell density at all levels, and excellent correlation was observed (Spearman's correlation analysis: p < 0.001, rho = 0.786, 95% CI: [0.765–0.806]). Figure 1 showed cell densities according to the SUN grade. Increases in cell density were observed with increases in grade (Kruskal–Wallis one-way analysis of variance: *p* < 0.001, in all SUN grade comparisons).

**Table 1:**
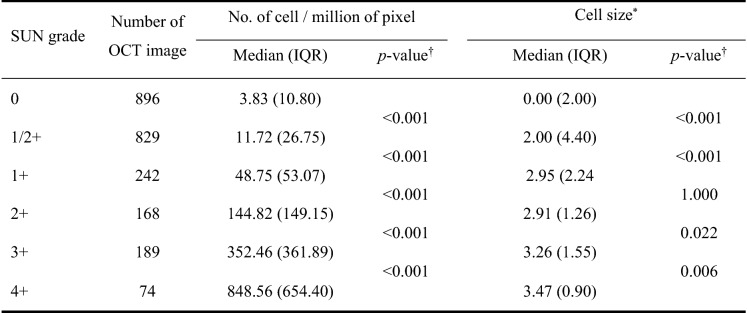
Comparison of cell density and cell size in anterior segment SD-OCT images in each grade.

*IQR* interquartile range.

*Continuous high reflective pixel area in the anterior chamber.

^†^p value of Wilcoxon signed-rank test adjusted by Bonferroni method.

### Comparison of eccentricity based on diagnosis

1156 images were classified as SUN grade higher than 0+. We separated 18,108 cells from these images and calculated the eccentricity. The number of cells were 13,242 for post-surgical inflammation, 2937 for uveitis, and 1929 for vitreous hemorrhage. The eccentricity according to diagnosis were 0.857 ± 0.059 for post-surgical inflammation, 0.866 ± 0.062 for uveitis, and 0.873 ± 0.069 for vitreous hemorrhage. In the Kruskal–Wallis chi-squared test, significant differences were observed (p < 0.001), and there were significant differences between the three groups in the post hoc test (Bonferroni method, p < 0.001).

### Comparison of images from above and below

**Figure 2:**
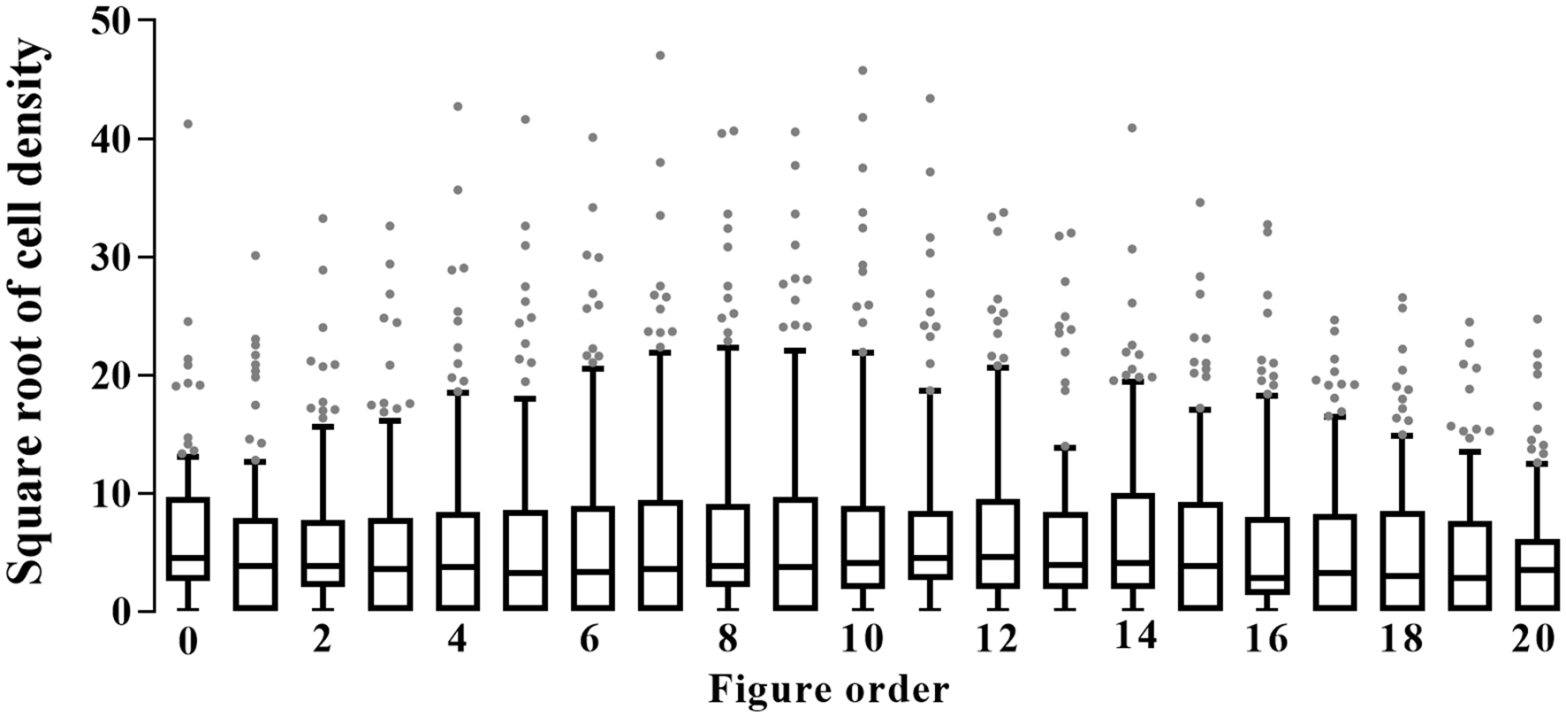
Cell density according to the location of imaging. Images 0 and 20 refer to the top and bottom of the anterior chamber, respectively. There were no significant differences in cell density in all areas of the anterior chamber (*p* = 0.2773, Kruskal–Wallis chi-squared test). The boxes indicate the median and interquartile range, and the whiskers indicate 10–90 percentile.

We investigated whether the cell density differed depending on the location of imaging. As images were taken from top to bottom in turn, image 0 was taken from the top of the cornea, and image 20 was taken from the bottom. There were no significant differences between the groups (*p* = 0.2773, Kruskal–Wallis chi-squared test) (Fig. 2).

## Discussion

Anterior chamber cell analysis using a slit lamp microscope is the most widely used evaluation tool in clinical ophthalmology practice^1^. However, the technique is associated with several problems. First, the grades are influenced by the width and brightness of the light^4^. In addition, the values obtained differ in each measurement because it is a subjective tool^3^. In addition, the grades have unequal intervals, and all grades 4 and above are rated the same. Therefore, there have been several attempts to measure anterior chamber inflammation objectively.

As anterior chamber cells can be clearly distinguished in anterior segment SD-OCT, many groups have attempted to image and quantify the cells using this method. Agarwal et al.^5^, who first analyzed anterior chamber cells by an automated method using OCT in 2008, were able to measure the cells effectively even with cornea edema and demonstrated a good correlation with SUN grades. In 2013, Li et al.^6^. set the threshold by adding twice the standard deviation to the average of the background noise of air to distinguish between noise and cells, and defined two or more pixels as cells. Other study suggested that anterior chamber cells can be measured simply by counting hyperreflective pixels^15^. Sharma et al.^8^. identified cells using their ImageIQ program; they defined hyperreflective pixels as cells based on a size between 3 and 50, and showed a good correlation with SUN grades. Although these studies have demonstrated the usefulness of anterior segment OCT, no automated analysis programs are commercially available.

It is possible to differentiate the anterior chamber cells from some images with the previously published method. However, several problems were found in other images. First, the brightness and exposure differed for each image, and excessive brightness or darkness led to difficulties in distinguishing the cells. Second, the threshold for distinguishing cells and background noise was incorrect. Third, artifacts often occurred in the OCT images, but there are no practical tools available for artifact removal. Therefore, although the existing method is theoretically suitable for analyzing clear images, noisy and distorted real-world images could not be analyzed with the method.

**Figure 3:**
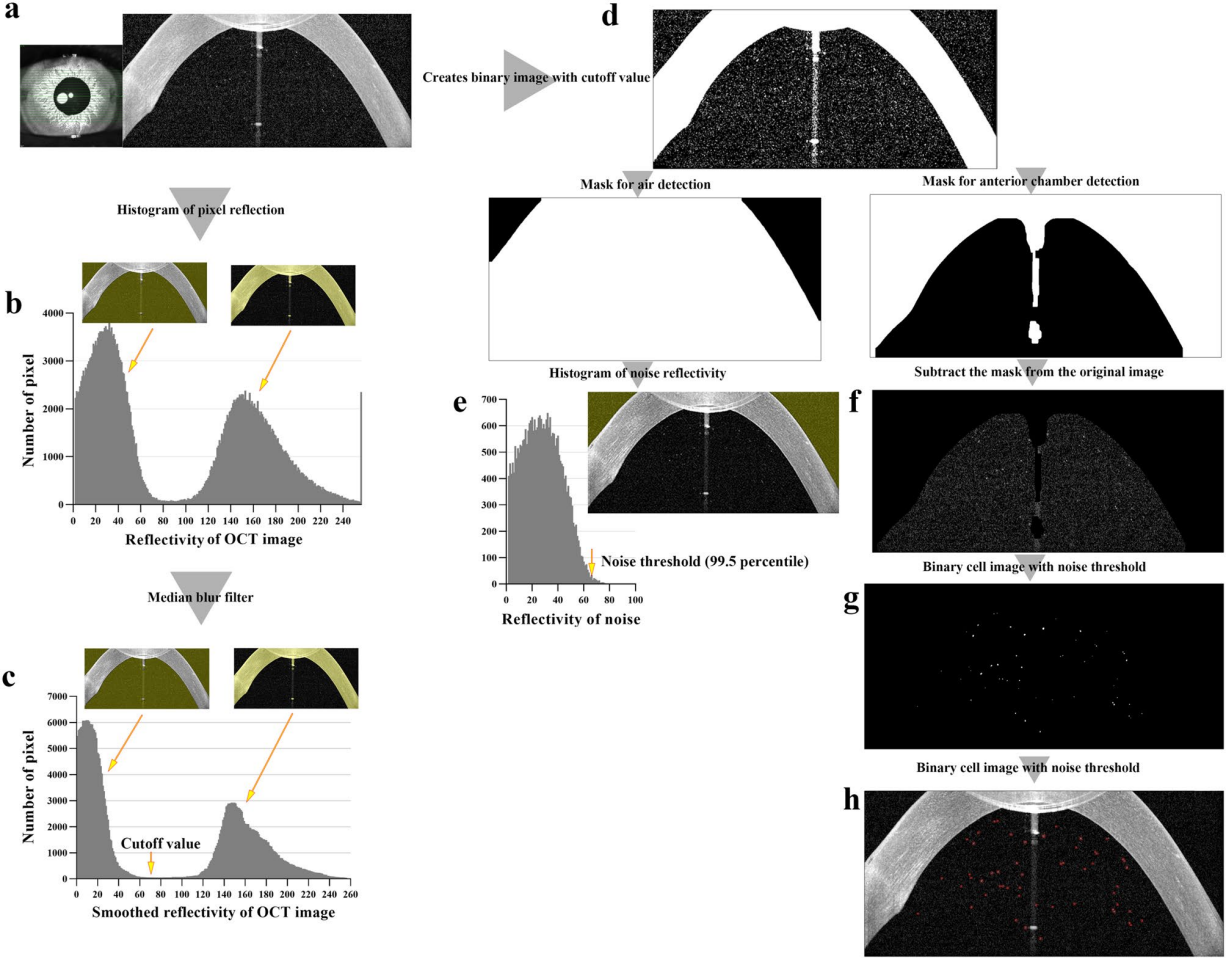
Schematic diagram of the method applied in this study. **a** The original image. Right image shows the current examination location with a bold green line, and left image shows air, cornea, and anterior chamber in order. The hyperreflective pixels in the anterior chamber are cells. Inverted artifacts in the cornea vertex and reflection artifacts in the center of the image are shown. **b** Histogram created using the entire pixel reflection. Noise-related pixels are clustered between 0 and 60, and hyperreflective pixels caused by the cornea, iris, and artifacts are clustered between 100 and 255. **c** After image smoothing with median blur, the histogram has more prominent two peaks and more apparent differences. **d** Generation of a mask corresponding to the cornea and artifacts. **e** Hyperreflective pixels in the air are referred to as noise. The histogram of these pixels does not fit the normal distribution. **f** Anterior chamber image after subtracting the cornea and air. **g** Binary image created based on the noise threshold. **h** The anterior chamber cell location was overlaid with color in the original image to verify the program was operating correctly.

We devised a few methods to solve this problem in this study. First, histograms were used as a method to segment the cornea regardless of differences in brightness. All anterior segment SD-OCT images consisted of anterior chamber with low reflectance and cornea with high reflectance. Consequently, the cornea could be distinguished by selecting an appropriate point between the two peaks (Fig. 3b, c). Second, in previous studies, a method of doubling the standard deviation from the average of the background noise was used to determine the cutoff for background noise^6,7^. In this study, the 99.5th percentile was used as a nonparametric method, and the cells could be effectively distinguished (Fig. 3e). Finally, median filter was used in this study to remove artifacts, and it was possible to remove large-sized artifacts effectively.

Consistent with our expectations, cell density increased significantly according to SUN grades measured clinically in this study (Fig. 1). Previously, there have been a report that more cells were seen in the lower part of the anterior chamber than in the upper part^6^. However, in this study, no differences in cell density were observed according to location within the chamber. This discrepancy may have been due to differences in the target group because the previous study targeted quiescent uveitis. Further studies regarding this issue are required.

The cells found in the anterior chamber are primarily divided into RBCs and WBCs. In addition, the type of WBCs appearing in the anterior chamber depends on the cause of the inflammation. In sarcoidosis^16^, tuberculosis^17^, and Vogt–Koyanagi–Harada^18^, monocytes appear in the anterior chamber, but neutrophils are predominant in toxic anterior segment syndrome^19^, infectious endophthalmitis^20^, and HLA-B27-associated uveitis^21^. As the types of blood cells vary according to disease, identifying these cells in a noninvasive manner can be helpful for both diagnosis and treatment. Previous studies using OCT reported that monocytes and neutrophils have different reflectance distributions and axial lengths, thereby making it possible to differentiate between blood cell types^7^.

However, we found two problems to discriminate WBCs using OCT. First, as shown in Table 1, the size of cells tended to increase when more cells were present in the anterior chamber. We assumed that cells tend to attach to each other if cell density was increased. Therefore, it would be necessary to adjust the process using blood-cell density for cell differentiation based on cell size. Second, the resolution was too low for determination of blood-cell size; RBCs are ~ 6 μm in diameter and lymphocytes are ~ 7 μm, whereas the resolution of OCT is 3.9 μm, which is about half the size of cells. Third, as the brightness differed between OCT images, the reflectance distribution was different in each image. In addition, the reflectance distribution did not fit the normal distribution, and was not normalized by the conventional conversion method. Therefore, nonparametric statistical methods should be used. In conclusion, this study had limitations for differentiation of blood cell types based only on the reflection distribution and axial length.

The greatest difference in shape between the two types of blood cells is that RBCs are flat disks, while WBCs are close to spherical in shape. Based on these morphological differences, we introduced the concept of eccentricity, which can be determined using the circumference and area of the cell. It is easy to determine the perimeter and area of a cell using image analysis software. If a cell is closer to a circle, the circumferential length will be shorter than the area and the eccentricity will be close to zero, while if it has an elongated elliptical shape, eccentricity will be close to 1. In theory, the eccentricity would be high for the flat side of a RBC and low for the round top of a RBC. Therefore, as the number of RBCs increases, the average eccentricity increases with increases in the standard deviation in the image, as the RBCs are photographed in various orientations. In this study, the eccentricity of the vitreous hemorrhage group in which the cells were mostly RBCs was significantly higher than other groups. Eccentricity is an index that could be used to evaluate the shapes of cells and is useful for differentiation of cell type as a supplement to the reflectance distribution and axial length.

**Figure 4:**
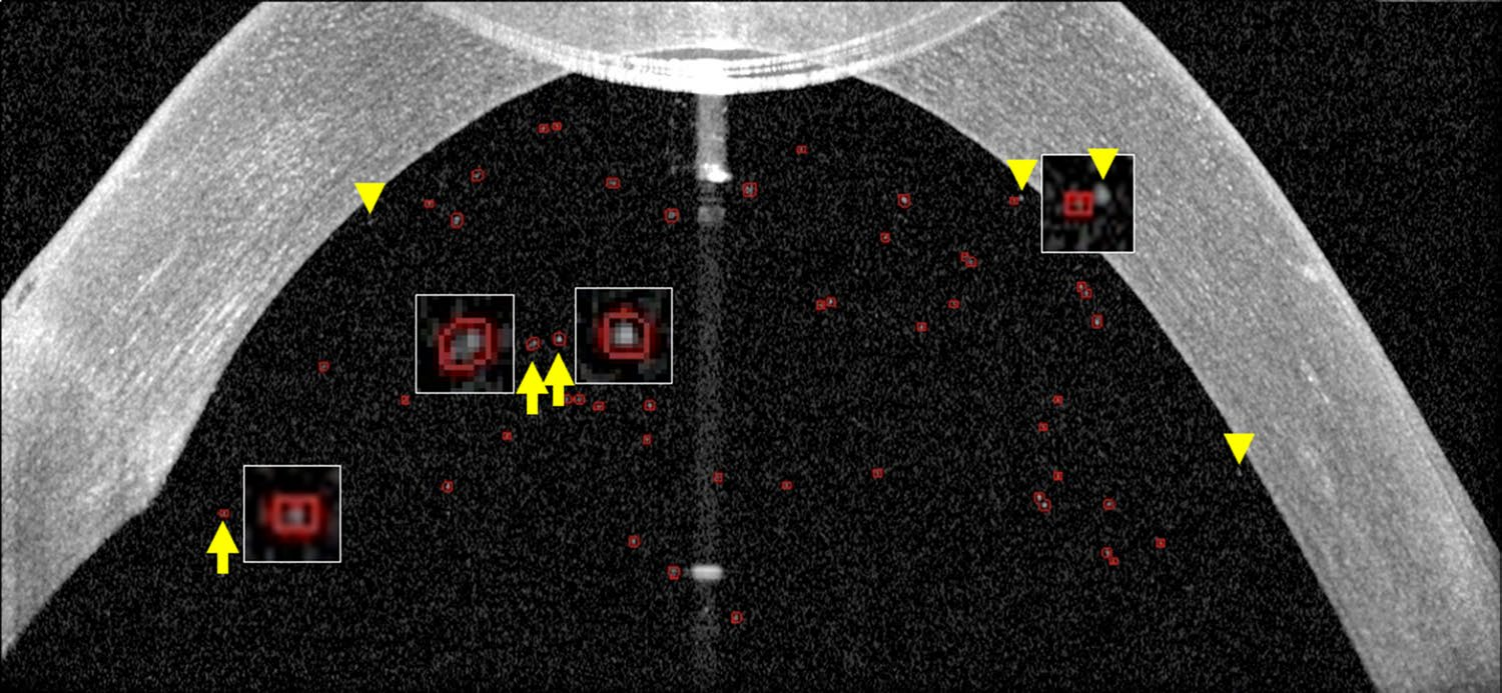
Various shape cell images. When one pixel is recognized as a cell, the area is recognized as 0 because of openCV limitation (left arrow). Large oval cells (middle arrow) and small round cells (right arrow) are also observed. Some cells are not recognized because these are too close to the cornea and covered by a mask (arrowheads).

This study had some limitations. First, the resolution of OCT was 3.9 μm, which is about half the size of the cells, and so the cell shape could not be completely captured. The eccentricity in this study was quite high because the cells were identified as angled pixels (Fig. 4). In addition, due to the limitation of the OpenCV package, the area was sometimes measured as 0 even though a cell was present (Fig. 4). Therefore, improvement of the software is necessary. Second, the presence of too many cells in the anterior chamber, such as in total hyphema, creates difficulties in distinguishing between the cornea and anterior chamber, resulting in part of the anterior chamber being classified as cornea or artifact in the mask. Therefore, the measured area of the anterior chamber is smaller than in reality, and the measured cell count is less than the actual count; however, cell density serves as a useful clinical indicator. Third, cells attached to the cornea or iris were not distinguished (Fig. 4). Forth, we could not discriminate attached cells because of poor SD-OCT resolution. However, these attached cells probably appear as a single cell even in a slit lamp examination and may affect SUN grading system too. Fifth, the software sometimes misrecognized noises as cells when the images were too dark. And the cornea was also misclassified as cells in the dark images. Finally, we analyzed data from a small number of patients, and the diagnosis was biased; only one patient with vitreous hemorrhage, six patients with uveitis. The results could be affected by selection bias. Thus multicentric studies with a larger number of eyes are needed to confirm the effectiveness of this study.

With improvements in computing power and the development of new software, image processing that would have been difficult previously has become both easy and inexpensive. Python is a useful programming language for image processing as it is distributed free of charge and has many useful packages and a very high degree of freedom. Extensive diagnostic and therapeutic innovations in the ophthalmic field are expected because of rapid developments in convolutional image processing and the introduction of highly flexible software, such as Python. If anterior chamber cell differentiation using anterior OCT becomes feasible, it will help to distinguish between inflammation and infection, and to establish treatment directions.

In conclusion, automated anterior chamber cell measurement using anterior OCT was rapid, highly accurate, and strongly correlated with SUN grade. Further research is necessary to confirm whether the newly presented concept of eccentricity for anterior chamber cell differentiation will be useful as an indicator for evaluating cell shape.

## Methods

### Study design

This study was approved by the institutional review board of Gyeongsang National University, Changwon Hospital (GNUCH 2020-07-009-002) and adhered to the tenets of the Declaration of Helsinki. The requirement for obtaining informed patient consent was waived from the institutional review board of Gyeongsang National University Changwon Hospital due to the retrospective nature of the study. A retrospective chart review of patients diagnosed with hyphema and post-surgical inflammation at the hospital between March 1, 2017, and December 31, 2019, was performed.

### OCT measurements

An expert performed anterior segment OCT scans using a Spectralis OCT device with an anterior segment module (Heidelberg Engineering, Heidelberg, Germany), which consists of an add-on lens and dedicated software. This system acquires 40,000 A-scans per second with an axial resolution of 3.9 m/pixel in tissue and a transverse resolution of 5.7 m/pixel. Anterior chamber images were acquired using the cornea setting. Twenty-one cross-sectional images were taken at a time, and the interval between images was 278 μm. Each cross-sectional image subtended an angle of 20° (Fig. 3a). All scans were acquired using automated real-time (ART), which was set at 20 frames (i.e. each image comprised 20 averaged B-scans).

### Image acquisition protocol

Anterior chamber images were taken using Heidelberg Eye Explorer (HEYEX) software (version 1.6.8; Heidelberg Engineering). Images were acquired and saved by an automated method using code written in Python to create an image set. During acquisition, the grayscale images were saved in BMP file format.

### Distinguishing air in OCT images

OCT images were divided into air, cornea, and anterior chamber (Fig. 3a). The air and the anterior chamber appeared as hyporeflective pixels, while the cornea appeared as distinct hyperreflective pixels. The following procedure was determining the threshold for distinguishing the cornea from the air and the anterior chamber. A histogram was created using the entire pixel reflection. Of the two peaks seen in the histogram, the left pixels were related to the anterior chamber and air, and the right pixels were related to the cornea (Fig. 3b). The OCT image was processed with a median blur to reduce the irregularity in the histogram (Fig. 3c). Noise and the cornea were distinguished by gradient descent method as followed. At each point on the histogram, calculate the average of 11 bins, including 5 bins on the left side and 5 bins on the right side. We repeated this operation and moved to the right until finding a zero gradient point. If the average was higher than the right side average (positive gradient) and the average was lower than the left side average (negative gradient), the point was zero gradient. The point was determined as the cutoff value. After converting the original image to a binary image with the cutoff value, we created two masks for air and anterior chamber detection (Fig. 3d).

### Generation of a mask for image artifacts

The anterior chamber cell was relatively small in size, but the artifacts were relatively large and tended to occur from top to bottom of the OCT images. To remove the artifacts, a mask was generated with a median blur using a 21 × 21-pixel kernel. This mask removed large hyperreflective pixels that did not pass through the 21 × 21-pixel kernel, and small hyperreflective pixels remained unaffected (Fig. 3d). Subsequently, a median blur was created using a 3 × 3-pixel kernel. Finally, the hyperreflective pixels that remained in the median blur using a 3 × 3-pixel kernel but that were removed in the mask using the kernel of 21 × 21-pixels were defined as cells. In addition, to remove reflection artifacts that appeared in a straight line from top to bottom, the 16 shades in the same column were defined as noise and were removed.

### Determining the cutoff values of noise and anterior chamber cells

In theory, there should be no reflection in air, so the hyperreflective pixels of air are referred to as noise. The histogram of the noise is shown in Fig. 3e. The histogram did not fit a normal distribution in the Shapiro test, and the distribution was severely skewed in some of the images. Therefore, a nonparametric method should be used to decide the noise threshold. We tried several nonparametric methods, and we found that the noise could be effectively eliminated when using the 99.5th percentile. After subtracting the cornea and air from the original image (Fig. 3f), the resulting image was converted to a binary image using the noise threshold (Fig. 3g).

### Processing the cell images

Perimeters and areas of each cell were calculated from the final binary image (Fig. 3g). In addition, the eccentricity was calculated by assuming that the cell was an ellipse. Finally, the original image was overlaid with the boundary line identified in the binary image to check for errors (Fig. 3h). The cell density was defined that the number of cells divided by the anterior chamber area (number of cells/million pixel). We compared the cell density with SUN grade.

### Formula for estimation of eccentricity

Assuming that the cell is an ellipse with a long axis length *b* and a short axis length *a*, the area *S* and perimeter *L* of the ellipse can be approximated by the following formula:

$$\begin{aligned} S & =\uppi ab \quad (a < b) \\ L & \approx 2\uppi \sqrt {\frac{1}{2}\left( {a^{2} + b^{2} } \right)} \\ \end{aligned}$$

The eccentricity *e* is defined as follows:

$$e = \sqrt {1 - \frac{{a^{b} }}{{b^{2} }}} \quad (a < b)$$

In this equation, the eccentricity can be derived from the area and perimeter of the ellipse.

$$e = \sqrt {1 - \frac{{L^{4} - 8\uppi ^{2} S^{2} - L^{2} \sqrt {L^{4} - 16\uppi ^{2} S^{2} } }}{{8\uppi ^{2} S^{2} }}}$$

Because of OpenCV software limitation, one pixel-sized cell area is measured as zero (Fig. 4). Thus small-sized cells result in bias of eccentricity. The perimeters of small cells are overestimated because of pixel-based data, resulting in overestimated eccentricity. In this study, the eccentricities were calculated from 3 or larger size cells.

### Software

In this study, Python version 3.7 was used for image analysis with the associated packages NumPy version 1.17.2 and OpenCV version 4.2.0. Statistical analysis was performed using R version 4.0.0. For the nonparametric test, the Kruskal–Wallis chi-squared test was used, and Dunn’s test with the Bonferroni method was used for post hoc analysis. In all analyses, *P* < 0.05 was taken to indicate statistical significance.

## Data Availability

Data supporting the findings of the current study are available from the corresponding author upon a reasonable request.
